# The UK Functional Assessment Measure (UK FIM+FAM): Psychometric Evaluation in Patients Undergoing Specialist Rehabilitation following a Stroke from the National UK Clinical Dataset

**DOI:** 10.1371/journal.pone.0147288

**Published:** 2016-01-29

**Authors:** Meenakshi Nayar, Roxana Vanderstay, Richard J. Siegert, Lynne Turner-Stokes

**Affiliations:** 1 Department of Palliative Care Policy and Rehabilitation, Kings College London, Faculty of Life Sciences and Medicine, London, United Kingdom; 2 Regional Hyper Acute Rehabilitation Unit (RHRU), Northwick Park Hospital, London, United Kingdom; 3 School of Public Health and Psychosocial Studies and School of Rehabilitation and Occupational Studies, Auckland University of Technology, Northcote, Auckland, New Zealand; University of Glasgow, UNITED KINGDOM

## Abstract

The UK Functional Assessment Measure (UKFIM+FAM) is the principal outcome measure for the UK Rehabilitation Outcomes Collaborative (UKROC) national database for specialist rehabilitation. Previously validated in a mixed neurorehabilitation cohort, this study is the first to explore its psychometric properties in a stroke population, and compare left and right hemispheric strokes (LHS vs RHS). We analysed in-patient episode data from 62 specialist rehabilitation units collated through the UKROC database 2010–2013. Complete data were analysed for 1,539 stroke patients (LHS: 588, RHS: 566 with clear localisation). For factor analysis, admission and discharge data were pooled and randomised into two equivalent samples; the first for exploratory factor analysis (EFA) using principal components analysis, and the second for confirmatory factor analysis (CFA). Responsiveness for each subject (change from admission to discharge) was examined using paired t-tests and differences between LHS and RHS for the entire group were examined using non-paired t-tests. EFA showed a strong general factor accounting for >48% of the total variance. A three-factor solution comprising motor, communication and psychosocial subscales, accounting for >69% total variance, provided acceptable fit statistics on CFA (Root Mean Square Error of Approximation was 0.08 and Comparative Fit Index/ Tucker Lewis Index 0.922/0.907). All three subscales showed significant improvement between admission and discharge (p<0.001) with moderate effect sizes (>0.5). Total scores between LHS and RHS were not significantly different. However, LHS showed significantly higher motor scores (Mean 5.7, 95%CI 2.7, 8.6 p<0.001), while LHS had significantly lower cognitive scores, primarily in the communication domain (-6.8 95%CI -7.7, -5.8 p<0.001). To conclude, the UK FIM+FAM has a three-factor structure in stroke, similar to the general neurorehabilitation population. It is responsive to change during in-patient rehabilitation, and distinguishes between LHS and RHS. This tool extends stroke outcome measurement beyond physical disability to include cognitive, communication and psychosocial function.

## Introduction

Stroke is a leading cause of disability in the United Kingdom with over 152,000 strokes being reported each year [1]. Stroke patients are a diverse and heterogeneous group. Clinical syndromes such as language difficulties tend to be associated with left hemispheric strokes, while right hemispheric strokes have been linked with neglect [2] and impairments in integrative and interpretive aspects of cognition [3]. These disabilities can have a substantial negative impact on the independence of patients.

Disability measures such as the Barthel Index (BI) [4] and the Functional Independence Measure (FIM™) have been widely used in the context of a stroke [5–7]. However, although they capture the level of independence in the basic activities of daily living, they focus largely on physical function, and clinicians often find them lacking in the assessment of more subtle aspects of cognitive and psychosocial function [8].

The Functional Assessment Measure (FAM) was developed in the early 1990s by the Santa Clara Valley Medical Center in California, US [9] for use in patients with traumatic brain injury. The FAM does not stand alone, but extends the 18-item FIM, by adding 12 items that focus on cognitive and psychosocial function [9]. The tool was adapted for use in the UK in the late 1990s and refined to address some of the weaknesses in the original version [8]. It has been validated for traumatic brain injury and general neurorehabilitation population [10]. The UK FIM+FAM now forms the principal outcome measure for the UK national database for specialist rehabilitation in patients with complex disabilities [11] (the UK Rehabilitation Outcomes Collaborative (UKROC)). It has been used in other countries in Europe (notably Spain), South America, Australasia, Iran and Japan; and has also been found to perform reliably in several other languages [12,13,14].

As many of the cognitive and psychosocial items are relevant to the stroke population, a question frequently asked of the UKROC helpline is whether the UK FIM+FAM has been specifically validated for use in stroke or not. It is therefore pertinent to explore a) whether its psychometric and scaling properties are the same in stroke patients as in traumatic brain injury and general neurorehabilitation populations, b) if it is responsive to the changes that occur during in-patient rehabilitation and c) whether or not it identifies (in a broad sense) the differences in cognitive, communicative and psychosocial function that may be expected to arise from strokes affecting different areas of the brain.

A recent study from Japan has examined the reliability and concurrent validity of the UK FIM+FAM (in Japanese translation) in stroke patients [13]. However, systematic review of literature revealed that the factor structure and responsiveness of UK FIM+FAM have not yet been examined in a purely stroke population. This article therefore presents the first formal evaluation of these psychometric properties of the UK FIM+FAM in a stroke population. Specific aims of the study were as follows:

1. In Part 1:
  - To examine the factor structure (dimensionality and internal consistency) of the UK FIM+FAM in patients with complex disabilities undergoing inpatient specialist rehabilitation following a stroke
  - To determine its responsiveness to change in functional independence between admission and discharge for this patient population
2. In Part 2:
  - To examine the extent to which the UK FIM+FAM identified the anticipated differences in functional abilities between left and right hemisphere stroke patients.

## Methods

### Design, Participants and Setting

This article presents a cohort analysis of a national multi-centre sample of left and right hemisphere stroke patients who were admitted to inpatient specialist rehabilitation programmes in the UK during a 3-year period between May 2010 and April 2013.

In the UK, the majority of stroke patients will make a good recovery with the support of their local (Level 3) stroke rehabilitation services. A smaller number of patients have more complex needs that require expertise, equipment and facilities of a district (Level 2) or tertiary (Level 1) specialist rehabilitation centre. Typically, these services take a selected population of mainly younger stroke patients with a mixture of physical, cognitive, communicative and/or psychosocial difficulties. Detailed criteria for admission to such services are available on the British Society of Rehabilitation Medicine website [15]. Outcome evaluation in this group must take account of the full range of disabilities, rather than just physical function.

The UK Rehabilitation Outcomes Collaborative (UKROC) provides the national clinical database for specialist rehabilitation in the UK. Established in 2010 with funding from the UK National Institute for Health Research (Programme grant RP-PG-0407-10185), the UKROC database collates information on needs, inputs and outcomes of all the case episodes of in-patient specialist rehabilitation of those admitted to specialist rehabilitation (Levels 1 and 2) services in England. Other UK centres participate on a voluntary basis. A national training programme is in place to ensure that clinical teams are trained in the use of the UKROC tools and outcome measures.

The dataset consists of demographic information and process data, together with a hierarchical system of outcome measurement that includes the Barthel Index (at the simplest level), the FIM and the UK FIM+FAM (at the most detailed level) [11], rated on admission and discharge. At the start of data collection, services could choose which of these measures to report as an outcome measure, depending on the time that clinicians were willing/able to spend collecting the data [11]. Since April 2013, however, reporting of the UK FIM+FAM has been mandatory for all level 1 and 2 specialist rehabilitation services in England [10].

For the purpose of this analysis, we extracted all the case episodes for stroke patients in whom a full set of UK FIM+FAM data was collected on both admission and discharge. Subarachnoid haemorrhage was excluded because it often causes a diffuse injury and pattern of deficit, which is atypical within the usual stroke population. The UKROC dataset includes a field for primary localisation of brain injury, including left and right hemisphere, as well as bilateral, frontal brainstem and diffuse. This localisation is recorded by the treating clinical teams. The dataset does not include information on neuroimaging, so we cannot exclude the possibility that some of the localisation data was misreported.

Extracted data were transferred to Microsoft Excel for cleaning, and then analysed using the IBM Statistical Package for Social Sciences (SPSS version 21).

### Measures—The UK FIM+FAM

The UK FIM+FAM consists of 30 items [8]. Each item is rated on seven levels with a score ranging from 1—‘Total dependence’ to 7—‘Complete independence’. Nine items address basic self-care including bladder and bowel management; seven items address transfers and mobility; six items address communication, and nine items address cognitive and psychosocial function. Scores are rated by the multidisciplinary team, according to the published scoring manual, within 10 days of admission and within the last 7 days before discharge from the rehabilitation programme. Rating takes approximately 20–30 minutes depending on the complexity of the case and the experience of the team. Further detail regarding development of the UK version is detailed elsewhere [8], and specific information on scoring (including the scoring manual for the UK FAM items) may be found on our website [16].

### Analysis

The UK FIM+FAM generates ordinal data and there is continued debate about the approach to statistical analysis in this context. Some authors favour techniques based on Item Response Theory such as Rasch analysis [17] whilst others support initial evaluation using traditional psychometric approaches based on Classical Test Theory, such as factor analysis [10,18]. Even though they are based on parametric assumptions, principal components and factor analysis are widely used in this context and have generally been considered appropriate for the initial stage of exploring and describing the relationships among a large set of variables, even where assumptions of normality may not strictly hold [19]. In this paper we present a traditional psychometric analysis. We are in the process of exploring Rasch analysis, which will be presented for publication separately.

We debated carefully whether to use parametric or non-parametric statistical analysis. According to Altman and Bland 2009, rank methods are sometimes useful, but parametric methods are generally preferable as they provide estimates and CIs and generalise to more complex analyses, especially where data may have many possible values (ie, long-ordinal data) and samples are large [20]. Factor analysis already uses parametric assumptions, and for our primary analysis, we therefore used parametric techniques (t tests) for subscale analyses where ‘long ordinal’ data (range 28 to 96 points) approximated to a normal distribution. (For completeness, an equivalent analysis using non-parametric methods is provided in S1 and S2 tables, confirming that both methods gave similar results.) Non-parametric techniques were used in any event for item-level analyses that involved ‘short ordinal’ data (range 7 points) that were typically skewed, and so would not fulfil the assumptions of parametric techniques. To allow for multiple tests, the threshold for significance of two-sided P values was taken as 0.05/number of tests.

#### Part 1 analysis: psychometric evaluation

Overall dimensionality, internal consistency and responsiveness were examined for the whole stroke population (n = 1539).

**Dimensionality:** Factor structure of the UK FIM+FAM was examined first with an exploratory factor analysis (EFA), and then with a confirmatory factor analysis (CFA). In order to provide two samples that represented the full range of the scale, admission and discharge data were first pooled and then randomly divided into approximately equal samples using the random sample selection function within SPSS.

After establishing that the two samples were broadly equivalent in terms of demographics and total UK FIM+FAM scores, EFA was conducted on the first sample using a principal components analysis (PCA) with Varimax rotation. The Keyser Myer-Olkin test and Bartlett’s Test of Sphericity were used to ensure that the correlation matrix was suitable for factor analysis. The decision as to the number of factors to rotate was based on consideration of the number of factors with Eigenvalues >1.5 and visual inspection of the scree plot. These are well-established methods that usually provide clear, interpretable solutions and allow direct comparison with the results of both the previous factor analyses of the UK FIM+FAM [10,21,22].

CFA was conducted on the second sample using the AMOS software. AMOS is a visual statistical software specifically used for confirmatory factor analysis. AMOS stands for Analysis of Moment Structures [23]. The quality of the model fit was assessed with five indices: (i) chi-square, (ii) p value>0.5, (iii) chi-square/df, (iv) Root Mean Square Error of Approximation (RMSEA) and (v) CFI/TLI. RMSEA of between 0.08 to 0.10 provides a mediocre fit and below 0.08 shows a good fit. Comparative fit index/ Tucker-Lewis index CFI/TLI values range from 0.00 to 1.00 for the last three indices, best fit is 0.90 or higher values [24].

**Internal consistency:** Internal consistency in the total scale and resulting subscales was assessed using Cronbach’s Alpha.

**Responsiveness:** The responsiveness (change between admission and discharge) was evaluated using the group comparison at both subscale- and item-level. Significance of change within each subscale was tested for using paired t-tests. Cohen’s Effect Size was also calculated as the mean score difference between admission and discharge divided by the standard deviation of admission score. Item-level differences were tested using Wilcoxon signed rank test.

#### Part 2 analysis: Comparison of left and right hemisphere stroke functional characteristics

In the second part of our analysis, episodes were extracted for which a patient’s left or right hemisphere localisation had been clearly identified by the rating team (n = 1154). Between-group differences in UK FIM+FAM subscale scores were evaluated at both subscale- and item-level. Unpaired T tests were used to compare subscales and Mann-Whitney tests were used to compare item level data.

### Ethics

The UKROC database collates de-identified data as part of routine clinical practice and the programme registered as a Payment by Results Improvement Project. The analysis of this routinely-collected data is classed as service evaluation, which does not require research ethics permission in the UK.

## Results

**Fig 1:**
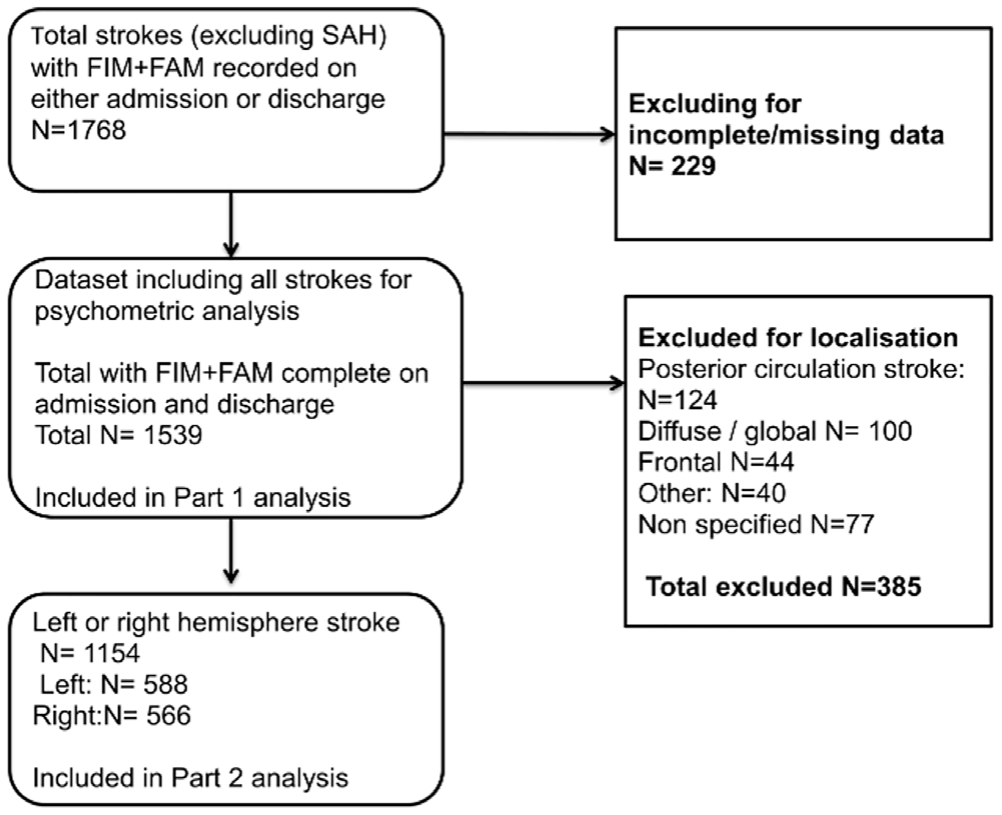
Flowchart of data extraction. Legend: Of a total stroke population of 1768), 1539 had complete UK FIM+FAM data and were included in the part 1 psychometric analysis; 1154 had been classified as left hemisphere (n = 588) or right hemisphere (n = 566) strokes and were included in the part 2 analysis.

The data selection and cleaning process is summarised in “Fig 1”. A total of 1768 stroke episodes were identified from units (n = 68) that routinely recorded the UK FIM+FAM for stroke patients during the data collection period. Of these, 1539 (87%) had complete UK FIM+FAM scores on both admission and discharge.

Table 1 shows the demographics for a) the total stroke population (n = 1768), b) the analysed stroke sample with complete UK FIM+FAM data (n = 1539), c) those in which the clinical teams had specified the stroke location as left hemisphere (n = 588) or right hemisphere (n = 566). No significant differences were found between any of the groups, suggesting that the various subgroups are reasonably representative of the whole stroke sample.

**Table 1:** Demographic characteristics for the various patient populations.

Parameter	Total strokes n = 1768	Analysed sample n = 1539	Left Hemisphere n = 588	Right Hemisphere n = 566
Age (years)(Mean SD)	58.2(16.3)	58.1(16.0)	58.7(16.4)	55.7(15.6)
Male: female ratio (%)	60:40%	60:40%	61:39%	59:41%
Length of stay (days)(Mean SD)	83.4(63.0)	80.7 (59.1)	78.1(55.1)	76.1(52.3)
**Aetiology: n (%)**				
Infarct	993(56.2%)	878(57.1%)	352(59.9%)	338(59.7%)
Haemorrhage	630(35.6%)	546(35.5%)	197(33.5%)	184(32.5%)
Other (or non-specified)	145(8.2%)	115(7.5%)	39(6.6%)	44(7.8%)

SD Standard deviation; n number.

After pooling the admission and discharge UK FIM+FAM scores and randomly splitting the sample into two groups, sample A consisted of 1550 UK FIM+FAM ratings and sample B of 1528. There were no significant between-group differences in age, gender ratio, length of stay or total UK FIM+FAM scores, confirming that the randomisation process had successfully delivered two approximately equal groups. Both datasets covered the full scoring range with less then 2% of ratings at the maximum (210) and minimum (30) score, confirming that there were no significant floor or ceiling effects in the sample.

### Part 1: Psychometric Analysis

#### Exploratory factor analysis

EFA was conducted on sample A. All items loaded reasonably strongly onto the first principal component with all 30 loadings >0.3. Inspection of the scree plot suggested a 3-factor solution with three principal components with Eigenvalues >1.5.

As a previous factor analysis in a general neurorehabilitation sample [10] suggested both a 2-factor solution and a 3-factor solution, both solutions were explored (see Table 2). Results showed:

- The 2-factor solution accounted for 64% of the variance and divided neatly into Motor (16 items) and Cognitive (14 items) subscales.
- The 3-factor solution accounted for 69% of the variance:
  - 15 out of 16 items again loading strongly on the first component reflecting the Motor function.
  - The 14 item cognitive subscale was split into two subscales:
    - Nine items loaded onto the second component (Psychosocial function).
    - Five items loaded on to the third factor (‘Communication’).

**Table 2:** Principal component loadings after Varimax rotation.

UK FIM+FAM item	Single component*	2-factor solution		3-factor solution		
		Motor	Cognitive	Motor	Psychosocial	Communication
**Eating**	0.482	0.614		0.601		
**Swallowing**	0.308	(0.446)				
**Grooming**	0.717	0.734		0.722		
**Bathing**	0.831	0.865		0.857		
**Dressing Upper Body**	0.788	0.842		0.835		
**Dressing Lower Body**	0.873	0.909		0.903		
**Toileting**	0.878	0.915		0.910		
**Bladder**	0.651	0.754		0.748		
**Bowels**	0.675	0.767		0.760		
**Bed transfers**	0.887	0.924		0.920		
**Toilet transfers**	0.887	0.925		0.921		
**Bath transfers**	0.773	0.869		0.865		
**Car Transfers**	0.712	0.831		0.828		
**Locomotion**	0.762	0.861		0.858		
**Stairs**	0.732	0.849		0.846		
**Community mobility**	0.499	0.649		0.644		
**Comprehension**	0.646		0.800			0.686
**Expression**	0.605		0.773			0.795
**Reading**	0.562		0.726			0.682
**Writing**	0.464		0.642			0.665
**Speech Intelligibility**	0.360		0.542			0.768
**Social interaction**	0.495		0.694		0.684	
**Emotional Status**	0.352		0.570		0.645	
**Adjustment**	0.610		0.755		0.809	
**Leisure activities**	0.607		0.655		0.575	
**Problem Solving**	0.695		0.769		0.749	
**Memory**	0.670		0.805		0.786	
**Orientation**	0.635		0.781		0.732	
**Concentration**	0.561		0.689		0.773	
**Safety awareness**	0.588		0.704		0.728	

Loadings < 0.5 were suppressed

*Pearson Item-total correlations for the single scale were all significant at p < 0.01

The 3-factor solution was considered the most promising model for stroke patients, as it accounted for 5% more of the variance and was readily interpretable. The only item that did not load onto any of these three components by >0.5 was ‘Swallowing’, which loaded weakly onto both the motor (0.433) and the communication (0.443) components. For subsequent analyses, it was included in the motor subscale on the basis of clinical relevance.

The internal consistency was high for the whole scale with Cronbach’s alpha = 0.96. Alpha coefficients for the Motor, Psychosocial and Communication subscales were 0.97, 0.93 and 0.88 respectively.

#### Confirmatory Factor Analysis

To determine the reliability of the hypothesised three-factor model yielded by EFA, the second randomly selected sample B (n = 1528) was examined using CFA. The model was specified to estimate each of the loadings on the three-factor hypothesised model (Table 2).

Modification indices for the following item pairs ‘eating and swallowing’, ‘Transfer bed and Transfer toilet’, ‘reading and writing’, ‘social interaction and emotional status’, ‘dressing upper and dressing lower’, ‘grooming and dressing upper’, ‘stairs and mobility’, ‘expression and speech’, ‘bladder and bowels’ all had large values suggesting a degree of overlap in item content. The model fit was further improved by allowing for covariance between the error terms of these pairs of items [23].

The fit statistics for the initial model was RMSEA = 0.115, CFI/TLI = 0.83/0.807. For the final model, the RMSEA was 0.080, CFI/TLI 0.922/0.907. These fit statistics for the final model met the criteria for mediocre but acceptable fit to the data. The final model approached the three-factor hypothesised structure of the UK FIM+FAM scale found in the present exploratory factor analysis, which was also the same as the structure previously reported in a general neurorehabilitation sample [10].

#### Responsiveness to Change

All UK FIM+FAM subscales showed significant improvement between admission and discharge (p < 0.0001) as shown in Table 3.

**Table 3:** Change in the UK FIM+FAM subscale scores from admission to discharge.

UK FIM+FAM item	Admission Mean (SD	Discharge(SD)	Mean diff	95% CI	T-test	P value	Effect size*
**All strokes**							
Motor	54.7 (27.0)	78.1 (26.9)	-23.4	22.5, 24.3	-51.0	<0.001	0.87
Cognitive	60.8 (20.5)	73.7 (17.5)	12.1	12.3, 13.5	-41.8	<0.001	0.63
Psychosocial	38.6 (13.7)	47.2(11.8)	8.7	8.2, 9.1	-37.9	<0.001	0.63
Communication	22.2 (8.7)	26.5 (7.3)	4.3	4.0, 4.5	-35.1	<0.001	0.49
**Left Hemisphere strokes**							
Motor	57.5 (27.4)	81.4 (25.2)	23.9	22.5, 25.3	-33.0	<0.001	0.87
Cognitive	57.4(20.4)	71.4 (17.9)	14.0	13.0, 14.9	-33.0	<0.001	0.69
Psychosocial	40.3 (13.1)	47.9 (11.6)	7.6	6.9, 8.2	-21.5	<0.001	0.58
Communication	25.8 (7.7)	29.0 (6.3)	3.3	2.9, 3.7	-17.6	<0.001	0.42
**Right Hemisphere strokes**							
Motor	51.9 (24.0)	75.6 (25.9)	23.8	22.4, 25.2	-33.0	<0.001	0.99
Cognitive	66.1(19.0)	76.9 (16.4)	10.8	9.9, 11.8	-30.2	<0.001	0.57
Psychosocial	38.5 (13.8)	47.5 (11.6)	9.0	8.4, 9.7	-26.0	<0.001	0.65
Communication	19.0 (8.5)	23.9 (7.7)	4.9	4.6, 5.3	-26.7	<0.0001	0.58

*Cohen’s Effect size–(mean score on discharge -mean score on admission)/Standard deviation of admission score. Interpretation: 0.2 = small, 0.5 = moderate, 0.8 = large

SD, Standard Deviation; CI, confidence Interval

**Fig 2:**
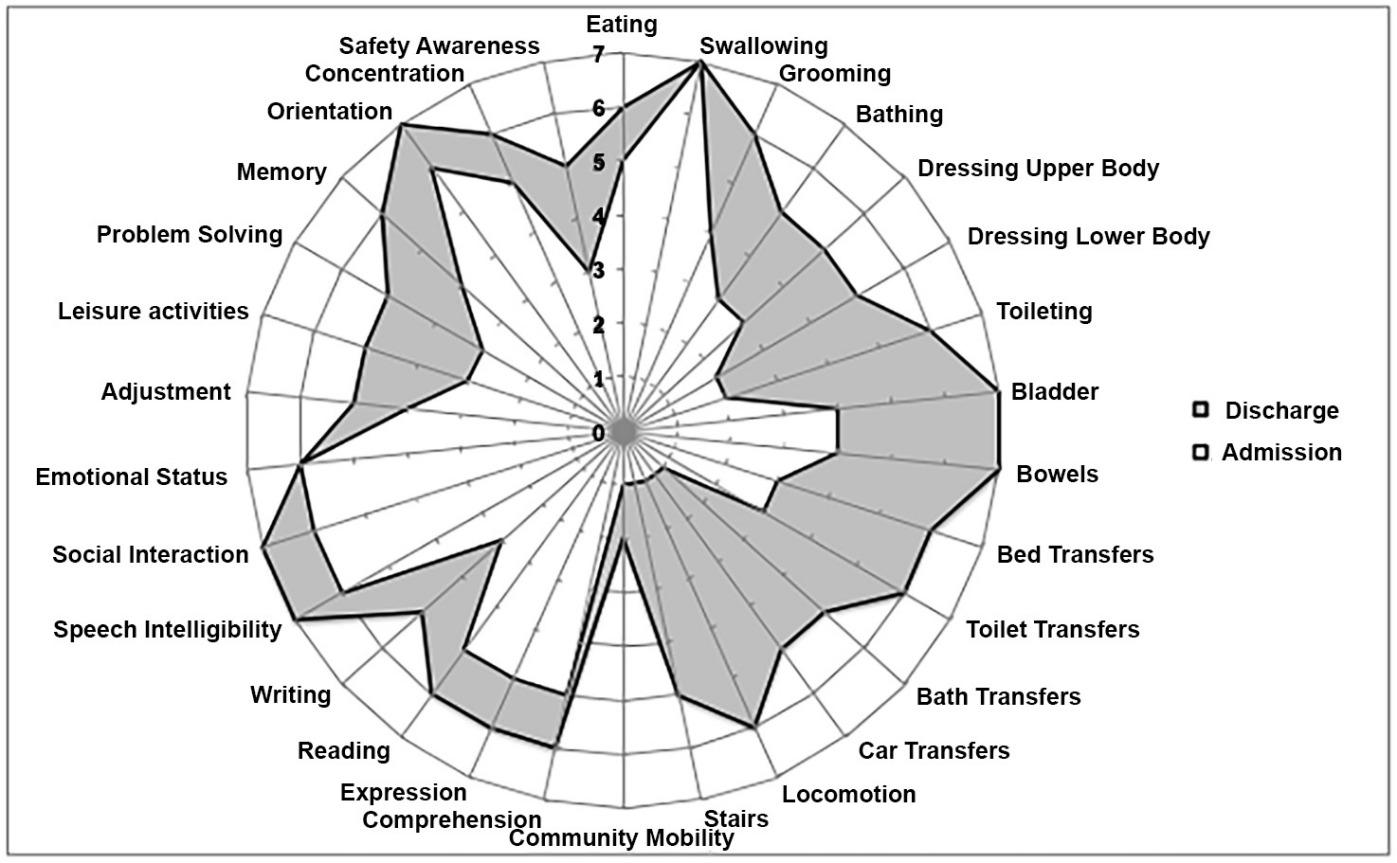
Composite radar chart of median item scores on admission and discharge for the whole stroke population. Legend: The radar chart (or “FAM splat”) provides a graphic representation of the disability profile from the data (n = 1539). Scale items are arranged as spokes of a wheel from 1 (total dependence) to 7 (total independence) run from the centre outwards. Thus a perfect score would be demonstrated as a large circle. This composite radar chart illustrates the median scores on admission and discharge. The shaded area thus represents the change in median score from admission to discharge.

The ‘composite radar chart’ “Fig 2” illustrates these changes at the individual item level. Although the largest changes were seen in the motor items (especially those reflecting mobility and continence), significant gains were seen for all items confirming the relevance of cognitive and psychosocial measurement in the stroke population.

### Part 2: Left versus Right Hemispheric Strokes

Table 4 shows the difference in UK FIM+FAM subscale scores between left and right strokes on admission. Overall, there was no significant difference in total UK FIM+FAM score between the left and right strokes; however the patterns of disability were different. After correcting for multiple tests, left hemisphere strokes showed significantly higher motor scores (Mean 5.7, 95%CI 2.7, 8.6 p<0.001), while left hemisphere strokes had significantly lower cognitive scores, primarily in the communication domain (-6.8 95%CI -7.7, -5.8 p<0.001). There was no significant group difference overall or in the psychosocial domain.

**Table 4:** Mean differences between left and right hemisphere strokes on admission.

UK FIM+FAM Scale	LeftMean (SD)	RightMean(SD)	Mean difference	95% CI	T-test	P value*
**Motor (range 16–112)**	**57.5(27.4)**	**51.9(24.0)**	**5.7**	**2.7, 8.6**	**3.7**	**<0.001**
*Cognitive(range 14–98)*	*57*.*4(20*.*4)*	*66*.*1(19*.*0)*	*-8*.*6*	*-10*.*9*, *-6*.*3*	*-7*.*4*	*<0*.*006*
Psychosocial(range 9–63)	38.5(13.7	40.3(13.1)	-1.8	-3.4, -0.3	-2.3	<0.02
*Communication(range 5–35)*	*19*.*0(8*.*5)*	*25*.*7(7*.*7)*	*-6*.*8*	*-7*.*7–5*.*8*	*-14*.*2*	*<0*.*001*
Total UK FIM+FAM	115.0(41.4)	117.9(36.2)	-2.9	-7.4, 1.5	-1.3	<0.20

*Two-tailed significance Threshold for significance: p<0.0125.

Statistically significant subscales which were higher in left-sided strokes are shown in bold and those which were higher in right-sided strokes are shown in italics.

**Fig 3:**
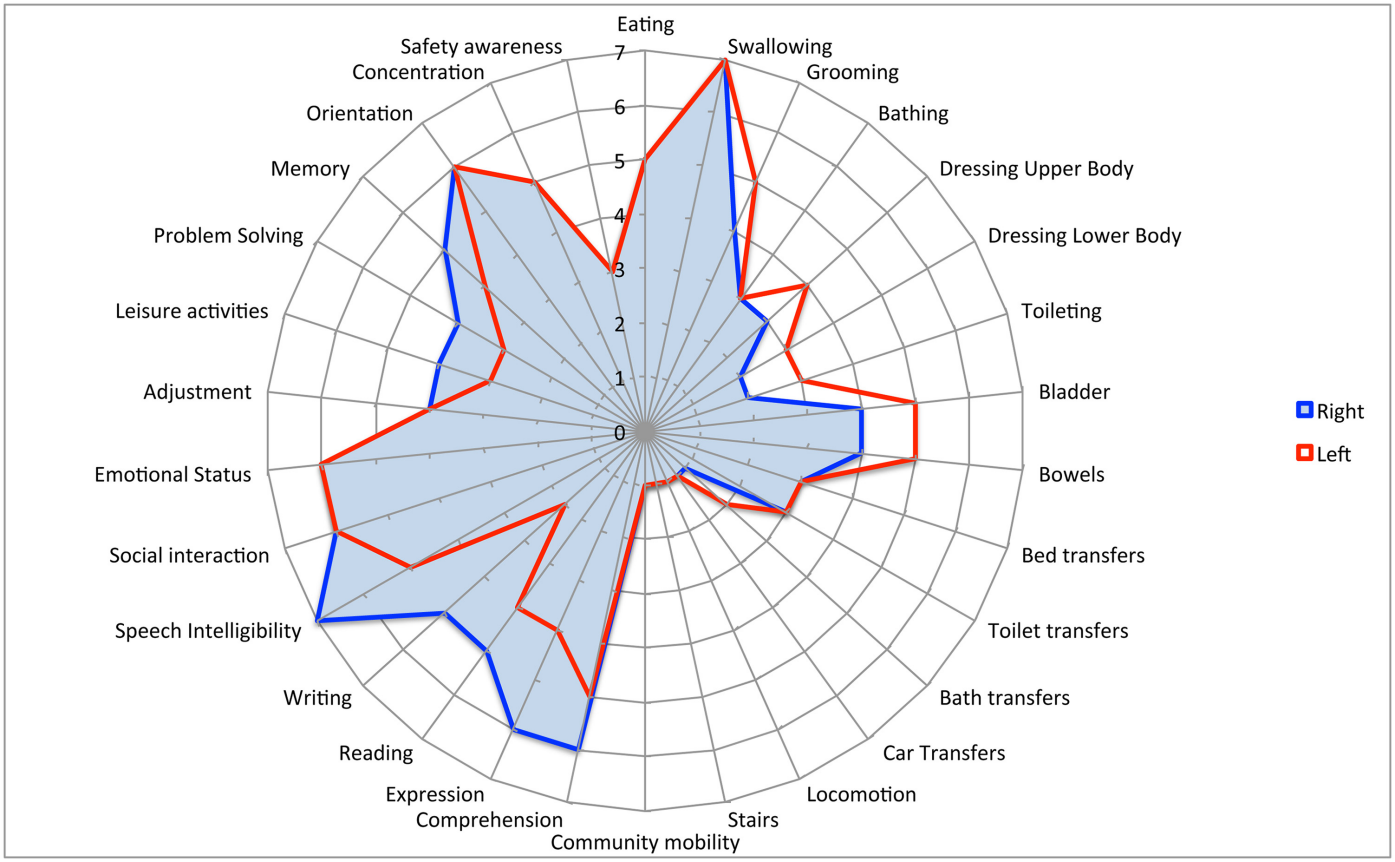
Composite radar chart of median item scores for Left and Right hemisphere strokes on admission. This composite radar chart illustrates the median scores for Left and Right hemisphere strokes. Left Hemisphere scores are shown in red, while Right hemisphere strokes are shown in blue. Left-sided strokes had higher scores for some motor items but lower scores for communication and some cognitive items.

The differences at item level shown in “Fig 3” were analysed and the results are provided in Table 5. Patients with right hemisphere stroke had significantly lower scores for dressing, toileting, bed and car transfers, locomotion and stairs; whilst patients with left hemisphere strokes had lower levels of all five communication items, memory and orientation.

**Table 5:** Item level differences between left and right strokes.

	Left Hemisphere Strokes					Right Hemisphere Strokes					Mann Whitney	
UK FIM+FAM item	Mean	SD	Median	IQR	Min-Max	Mean	SD	Median	IQR	Min-Max	z	p value*
Eating	5.16	1.63	5	5–7	1–7	5.08	1.66	5	5–7	1–7	-0.81	<0.415
Swallowing	6.05	1.63	7	5–7	1–7	6.04	1.63	7	5–7	1–7	-0.14	<0.887
Grooming	4.29	1.84	5	3–6	1–7	4.15	1.70	4	3–5	1–7	-1.36	<0.175
Bathing	3.45	1.87	3	2–5	1–7	3.16	1.67	3	2–4	1–7	-2.29	<0.022
**Dressing Upper Body**	**3.86**	**1.94**	**4**	**2–5**	**1–7**	**3.38**	**1.83**	**3**	**2–5**	**1–7**	**-4.22**	**<0.001**
**Dressing Lower Body**	**3.14**	**2.06**	**3**	**1–5**	**1–7**	**2.73**	**1.84**	**2**	**1–4**	**1–7**	**-3.19**	**<0.001**
**Toileting**	**3.48**	**2.34**	**3**	**1–6**	**1–7**	**2.99**	**2.14**	**2**	**1–5**	**1–7**	**-3.53**	**<0.001**
Bladder	4.22	2.54	5	1–7	1–7	3.86	2.46	4	1–7	1–7	-2.62	<0.009
Bowels	4.29	2.54	5	1–7	1–7	4.08	2.48	4	1–7	1–7	-1.52	<0.13
**Bed transfers**	**3.71**	**2.27**	**3**	**1–6**	**1–7**	**3.15**	**2.03**	**3**	**1–5**	**1–7**	**-4.05**	**<0.001**
**Toilet transfers**	**3.61**	**2.28**	**3**	**1–6**	**1–7**	**3.09**	**2.09**	**3**	**1–5**	**1–7**	**-3.82**	**<0.001**
Bath transfers	2.91	2.28	2	1–5	1–7	2.56	2.02	1	1–4	1–7	-2.11	<0.035
**Car Transfers**	**2.42**	**2.27**	**1**	**1–4**	**1–7**	**1.94**	**1.84**	**1**	**1–2**	**1–7**	**-3.20**	**<0.001**
**Locomotion**	**3.00**	**2.41**	**1**	**1–5**	**1–7**	**2.48**	**2.11**	**1**	**1–5**	**1–7**	**-3.62**	**<0.001**
**Stairs**	**2.28**	**2.20**	**1**	**1–4**	**1–7**	**1.72**	**1.67**	**1**	**1–1**	**1–7**	**-4.51**	**<0.001**
Community mobility	1.68	1.45	1	1–2	1–7	1.47	1.18	1	1–1	1–7	-2.72	<0.006
*Comprehension*	*4.26*	*1.91*	*5*	*3–6*	*1–7*	*5.55*	*1.51*	*6*	*5–7*	*1–7*	*-11.75*	*<0.001*
*Expression*	*3.70*	*2.11*	*4*	*2–6*	*1–7*	*5.55*	*1.73*	*6*	*5–7*	*1–7*	*-14.73*	*<0.001*
*Reading*	*3.55*	*2.11*	*4*	*1–5*	*1–7*	*4.62*	*2.31*	*5*	*2–7*	*1–7*	*-8.29*	*<0.001*
*Writing*	*2.79*	*1.97*	*2*	*1–4*	*1–7*	*4.08*	*2.50*	*5*	*1–7*	*1–7*	*-8.26*	*<0.001*
*Speech Intelligibility*	*4.66*	*2.16*	*5*	*3–7*	*1–7*	*5.94*	*1.55*	*7*	*5–7*	*1–7*	*-10.48*	*<0.001*
Social interaction	5.41	1.82	6	5–7	1–7	5.66	1.68	6	5–7	1–7	-2.60	<0.009
Emotional Status	4.95	2.09	6	3–7	1–7	5.11	1.99	6	4–7	1–7	-1.24	<0.215
Adjustment	3.97	1.91	4	2–6	1–7	4.11	1.88	4	3–6	1–7	-1.29	<0.196
Leisure activities	3.55	1.99	3	2–6	1–7	3.78	1.99	4	2–6	1–7	-1.95	<0.052
Problem Solving	3.44	1.90	3	2–5	1–7	3.67	1.97	4	2–5	1–7	-1.92	<0.054
*Memory*	*3.93*	*2.13*	*4*	*2–6*	*1–7*	*4.39*	*2.11*	*5*	*3–6*	*1–7*	*-3.71*	*<0.001*
*Orientation*	*4.89*	*2.19*	*6*	*3–7*	*1–7*	*5.36*	*1.99*	*6*	*4–7*	*1–7*	*-3.80*	*<0.001*
Concentration	4.87	1.83	5	4–6	1–7	4.57	1.91	5	3–6	1–7	-2.64	<0.008
Safety awareness	3.46	1.88	3	2–5	1–7	3.66	1.80	3	2–5	1–7	-2.41	<0.016

*Two-tailed significance Threshold for significance: p<0.002. Statistically significant items which were higher in left-sided strokes are shown in bold and those which were higher in right-sided strokes are shown in italics.

## Discussion

This first analysis of data from a large national cohort of stroke patients undergoing specialist in-patient rehabilitation demonstrated the scalability of UK FIM+FAM in this population. In addition to providing a single measure of overall functional independence, it also breaks down broadly into ‘Motor’ and ‘Cognitive’ components, and the latter separates further into a 9-item Psychosocial and a 5-item Communication component. The scale was responsive, all three subscales demonstrating highly significant change over the course of the rehabilitation programme. These findings confirm that the performance of the UK FIM+FAM in stroke patients is very similar to that in other groups. Turner-Stokes 2013 [10] reported a similar motor and cognitive factor structure in a general neurorehabilitation sample. This mirrored the findings of Hawley et al. 1999 [22] in their examination of the factor structure of the original US version of the FIM+FAM in patients with traumatic brain injury.

It also distinguished the disability profiles of right and left hemisphere strokes in a manner that resonates with clinical experience. Regardless of handedness, most individuals have left hemisphere dominance [25], and damage to the dominant hemisphere is frequently associated with difficulties with communication due to dysphasia. By contrast, patients with right hemisphere strokes tend to have relatively intact communication skills, but experience a range of cognitive and motor planning deficits (including left-sided neglect and motor dyspraxia) that impact their daily functioning. In our analysis, left hemispheric strokes were found to have significantly worse function than right hemispheric strokes on all aspects of communication, while the right hemisphere strokes had worse function in the domains of dressing and also some aspects of transfers and mobility. The results showed the expected differences between left and right hemispheric strokes, confirming that the FIM+FAM is sensitive to these differences.

With the exception of memory and orientation, there was no significant difference between left and right-sided stroke patients in the cognitive and psychosocial domains of the FIM+FAM, but both groups had significant deficits in these areas, which improved significantly during the course of rehabilitation. These findings confirm the importance of measuring aspects of cognitive and psychosocial function, in addition to physical disability, as part of routine outcome evaluation in stroke patients.

There has been considerable debate in the literature about the added value of the FIM+FAM over the FIM. Some authors have failed to show that the FAM items provide increased sensitivity at a statistical level compared with the FIM alone [26,27], and argue that the extended scale adds little benefit from a measurement perspective. On the other hand, an outcome measure used in the evaluation of clinical practice should reflect the full range of function that is targeted for treatment. From a clinical perspective, health professionals working in the context of complex brain injury frequently express dissatisfaction with the limited coverage of psychosocial function within the FIM. There is evidence that the FIM+FAM provides better coverage (albeit still incomplete) across the wider range of activities that reflect patients’ personal goals for treatment in rehabilitation [28]. Hall et al 1996 [29] demonstrated that FAM items could extend the ceiling of the FIM in the context of traumatic brain injury, and the findings presented here suggest that this may also be true for complex stroke patients.

The addition of 12 items certainly increases the time taken to rate the FIM+FAM, which may have resource implications, but some clinicians report that this extra time and effort enhances team communication in more subtle areas of function that are often missed in clinical practice, and that this is rewarded by a more holistic picture of clinical performance in complex disability [30]. We do not suggest that the FIM+FAM is suitable for all settings, and accept that the FIM may be adequate for many of the general stroke rehabilitation settings that predominate in large datasets, for example in the US. Nevertheless, the UK FIM+FAM may be considered as an option where teams wish to extend the range of outcome evaluation to cover a wider range of psychosocial function in patients with complex needs. It also offers the advantage of preserving the FIM for the purpose of comparison with other international datasets, as well as the availability of a further module for the evaluation of extended activities of daily living [31].

### Study Limitations

1. The study was carried out in a selected stroke population of mainly younger adults with complex needs. It cannot be assumed that the findings would necessarily be reflected in a more typical older stroke population. However, the findings would have relevance for other countries that offer specialist rehabilitation services for selected groups of stroke patients with more complex needs.
2. The data were recorded in the context of routine clinical practice and 13% of episodes had incomplete FIM+FAM data. Although the included sample was not significantly different from the total population with respect to demographics or total functional scores, we cannot exclude the possibility of sample bias.
3. Within the main subgroups of left and right hemisphere stroke, there will inevitably be a range of pathologies that may impact the outcome. For example, the Bamford classification [32] separates strokes into total and partial anterior circulation, and lacunar strokes that are known to carry different outcomes [33]. In this sample, we know that the proportions of infarcts to haemorrhage strokes were similar for both sides of stroke, but the UKROC dataset does not include the Bamford classification or equivalent. Hence, we cannot be certain that the groups were well-matched for pathological severity. That said, however, patients referred to specialist inpatient rehabilitation are a selected sample of patients with more complex disabilities, whose recovery trajectory is likely to be slower. Therefore, we would anticipate a relatively low proportion of small vessel lacunar strokes in this study population.
4. The factor analysis was carried out on a sample where admission and discharge data was pooled together and randomised. The population was therefore heterogeneous, which may partly explain why the CFA indices were acceptable but did not meet the strictest criteria. Heterogeneity could potentially have been reduced by restricting the sample to admission values only, but we deliberately used a broader sampling method to ensure data representation across the whole scale range.
5. Although the sample size exceeded the usual standards for factor analysis, and by pooling and randomisation of the samples, we reduced, as far as possible, the relationship between samples used for EFA and CFA, they cannot be said to be fully independent. The results of CFA therefore require confirmation in a fully independent sample.

In summary, despite the above-recognised limitations, this study provides confirmation that the UK FIM+FAM is a valid instrument for use as a measure of functional independence in stroke patients. Its scaling properties are broadly similar in this group to those previously reported in a general neurorehabilitation population and in traumatic brain injury. It demonstrates deficits in cognitive, communicative and psychosocial function that change during rehabilitation. In this study, the FIM+FAM differentiated between patients with left and right hemisphere stroke in a manner that resonates with clinical experience, thus suggesting that it is an appropriate tool to use in this population, especially where the clinical team wishes to extend outcome measure beyond the simple recording of physical disability and independence in basic activities of daily living.

## Supporting Information

S1 Table Change in the UK FIM+FAM subscale scores from admission to discharge. Alternative analysis using non-parametric statistics.
(PDF)

S2 Table Mean differences between left and right hemisphere strokes on admission. Alternative analysis using non-parametric statistics.
(PDF)
