# Supplementary material for: The UK Functional Assessment Measure (UK FIM+FAM): Psychometric Evaluation in Patients Undergoing Specialist Rehabilitation following a Stroke from the National UK Clinical Dataset
Source: PLoS One. 2016 Jan 29;11(1):e0147288. doi: 10.1371/journal.pone.0147288 (PMC4732596; doi:10.1371/journal.pone.0147288)
Supplement: S1 Table — Alternative analysis using non-parametric statistics. (PDF) [file pone.0147288.s001.pdf]

S1 Table: Change in the UK FIM+FAM subscale scores from admission to discharge.
Alternative analysis using non-parametric statistics

UK FIM+FAM item	Admission Median (IQR)	Discharge Median (IQR)	Wilcoxon z	P value	Effect size*
**All strokes**					
Motor	48(33-74)	85(57-101)	-32.73	<0.001	0.97
Cognitive	62(45-77)	77(62-88)	-31.02	<0.001	0.93
Psychosocial	39(28-50)	50(39-57)	-29.64	<0.001	0.91
Communication	23(16-30)	28(22-33)	-28.53	<0.001	0.95
**Left Hemisphere strokes**					
Motor	51(34-80)	89(62-103)	-20.28	<0.001	0.97
Cognitive	59(41-74)	75(59-87)	-19.73	<0.001	0.95
Psychosocial	39(28-50)	50(39-57)	-19.01	<0.001	0.94
Communication	19(12-26)	25(18-30)	-18.93	<0.001	0.97
**Right Hemisphere strokes**					
Motor	47(33-66)	81(55-98)	-20.02	<0.001	0.97
Cognitive	67(53-82)	81(66-91)	-18.18	<0.001	0.93
Psychosocial	41(31-51)	50(40-58)	-17.25	<0.001	0.91
Communication	27(21-32)	31(26-34)	-15.71	<0.001	0.95

Effect size was calculated by dividing number of positive difference scores by the total number of matched pairs. One half the number of ties were added to the numerator.

Reference

Grissom RJ, Kim JJ. Effect size for research. Univariate and Multivariate applications. 2nd Edition. New York, NY: Taylor and Francis. 2012
