# Supplementary material for: The UK Functional Assessment Measure (UK FIM+FAM): Psychometric Evaluation in Patients Undergoing Specialist Rehabilitation following a Stroke from the National UK Clinical Dataset
Source: PLoS One. 2016 Jan 29;11(1):e0147288. doi: 10.1371/journal.pone.0147288 (PMC4732596; doi:10.1371/journal.pone.0147288)
Supplement: S2 Table — Alternative analysis using non-parametric statistics. (PDF) [file pone.0147288.s002.pdf]

S2 Table: Mean differences between left and right hemisphere strokes on admission.
Alternative analysis using non-parametric statistics

UK FIM+FAM Scale	Left Median (IQR)	Right Median (IQR)	Mann Whitney z	P value*
**Motor (range 16-112)**	**51(34-80)**	**47(33-66)**	**-3.10**	**<0.002****
Cognitive (range 14-98)	*59(51-74)*	*67(53-82)*	*-7.01*	*<0.001*
Psychosocial (range 9-63)	39(28-50)	41(31-51)	-2.17	<0.030
Communication (range 5-35)	*19(12-26)*	*27(21-32)*	*-13.1*	*<0.001*
Total UK FIM+FAM	115(82-149)	115(92-143)	-1.25	<0.20

***Two-tailed significance** Threshold for significance: p<0.0125.

****Subscales which were significantly higher in left-sided strokes are shown in bold**.

Those which were significantly higher in right-sided strokes are shown in italics.
